# Anticoagulant action of low, physiologic, and high albumin levels in whole blood

**DOI:** 10.1371/journal.pone.0182997

**Published:** 2017-08-11

**Authors:** Margret Paar, Christine Rossmann, Christoph Nusshold, Thomas Wagner, Axel Schlagenhauf, Bettina Leschnik, Karl Oettl, Martin Koestenberger, Gerhard Cvirn, Seth Hallström

**Affiliations:** 1 Institute of Physiological Chemistry, Medical University of Graz, Graz, Austria; 2 Department of Blood Group Serology and Transfusion Medicine, Medical University of Graz, Graz, Austria; 3 Department of Pediatrics, Medical University of Graz, Graz, Austria; Monash University, AUSTRALIA

## Abstract

Albumin is the most abundant plasma protein. Critical illness is often associated with altered, predominately decreased, serum albumin levels. This hypoalbuminaemia is usually corrected by administration of exogenous albumin. This study aimed to track the concentration-dependent influence of albumin on blood coagulation in vitro. Whole blood (WB) samples from 25 volunteers were prepared to contain low (19.3 ± 7.7 g/L), physiological (45.2 ± 7.8 g/L), and high (67.5 ± 18.1 g/L) levels of albumin. Haemostatic profiling was performed using a platelet function analyzer (PFA) 200, impedance aggregometry, a Cone and Platelet analyzer (CPA), calibrated automated thrombogram, and thrombelastometry (TEM). Platelet aggregation-associated ATP release was assessed via HPLC analysis. In the low albumin group, when compared to the physiological albumin group, we found: i) shortened PFA 200-derived closure times indicating increased primary haemostasis; ii) increased impedance aggregometry-derived amplitudes, slopes, ATP release, as well as CPA-derived average size indicating improved platelet aggregation; iii) increased TEM-derived maximum clot firmness and alpha angles indicating enhanced clot formation. TEM measurements indicated impaired clot formation in the high albumin group compared with the physiological albumin group. Thus, albumin exerted significant anticoagulant action. Therefore, low albumin levels, often present in cancer or critically ill patients, might contribute to the frequently occurring venous thromboembolism.

## Introduction

Albumin, the most abundant plasma protein, has numerous functions in health. It contributes up to 80% of the total colloid osmotic pressure, transports drugs and endogenous compounds, acts as an effective plasma buffer, exhibits significant antioxidant potential, and maintains microvascular integrity [1–3].

A link between low serum albumin and an increase in morbidity and mortality has been shown [4]. For example, hypoalbuminaemia in hospitalized patients is associated with increased length of stay and higher complication rates [5]. Non-survivors of critical illness have lower serum albumin concentrations than survivors [6]. Therefore, exogenous albumin is widely used to correct hypoalbuminaemia. For example, patients with severe burn injury as well as cirrhotic patients have been shown to benefit from albumin therapy [7, 8].

Several studies suggest that albumin affects blood coagulation. However, the findings presented therein are equivocal. On one hand, albumin has been shown to exert anticoagulant action due to its capability to bind antithrombin, associated with enhanced neutralization of coagulation factor Xa [9], and its inhibitory effect on platelet aggregation [10]. Moreover, albumin infusion has been shown to decrease the patients’ coagulation competence during major surgery by using thrombelastometry measurements [11]. Furthermore, albumin infusion has been shown to decelerate the clot formation process compared to to hydroxyethyl starch or Ringer’s lactate infusion postoperatively [12].

On the other hand, albumin has been reported to exert procoagulant action. An anionic form of albumin, present in normal human plasma, is capable of inducing tissue factor (TF) generation in monocytes which is associated with activation of coagulation [13]. Moreover, hypercoagulability following albumin administration has been demonstrated in rabbits [14].

This in vitro study aimed to clarify whether addition of exogenous albumin shifts the haemostatic system towards a hyper- or a hypocoagulable state. All previous studies investigating the effects of albumin on coagulation were mainly performed in platelet poor plasma (PPP) samples or in purified systems containing washed platelets. In contrast, the present study was performed mainly in whole blood (WB) samples. While PPP contains the majority of the coagulation factors implicated in the coagulation process, WB includes i.a. monocytes and platelets which support coagulation. We herein examined the effects of low, physiologic, and high albumin levels in WB on platelet adhesion, platelet aggregation, and on thrombelastometry values, a method suitable for real-time assessment of clotting speed and strength [15]. Additionally, thrombin formation curves and F1+2 levels were measured in PPP samples.

It has recently been shown in cancer patients that reduced serum albumin levels are significantly associated with an increased risk of venous thromboembolism (VTE) and mortality [16, 17]. However, these studies did not reveal whether low serum albumin concentrations are a direct cause for VTE or just an associated marker. Experimental data from the present study may help to elucidate whether low albumin levels have to be considered as a risk factor or as an associated marker for VTE in critically ill patients.

## Material and methods

### Reagents and devices

Human serum albumin (HSA), HClO_4_, K_2_CO_3_, and HiTrap^®^ Blue High Performance were purchased from Sigma-Aldrich (Vienna, Austria). A HSA stock solution was prepared by dissolving 700 mg of albumin in 1 mL of 0.9% saline. Recombinant human TF thromboplastin (Innovin^®^) was obtained from Dade Behring Marburg GmbH (Marburg, Germany). The fibrin polymerization inhibitor GPRP (Pefabloc FG) was obtained from Pentapharm LDT (Basel, Switzerland). HSA plasma levels were determined on a spectrometer from Bio-Tek Instruments Inc. (Winooski, Vermont, USA) at wavelength 578 nm using the bromocresol green method; the respective reagents were from HUMAN (Wiesbaden, Germany). The Platelet Function Analyzer (PFA) 200 was from Siemens Healthcare Diagnostics (Vienna). All other chemicals were from Sigma (Vienna).

### Subjects

After approval of the appropriate institutional review board (ethics committee of the Medical University of Graz; 27–320 ex 14/15) and with written informed consent, a total of 25 healthy volunteers (14 male, 11 female) aged between 29 and 44 years were recruited. All volunteers denied taking any medication within the last two weeks which might influence coagulation. The volunteers did not suffer from renal or liver disease or coagulation disorders.

### Blood collection and preparation

Twenty five subjects were enrolled and the obtained blood was split in two sets of experimental batches to perform the different analyses. The WB measurements were performed within 3h of blood sampling. Blood sampling was performed in two consecutive steps for each subject. In the first step, nine mL of blood from the antecubital vein were collected into pre-citrated Vacuette^®^ marked tubes (Greiner Bio-one GmbH, Kremsmünster, Austria) containing 3.8% sodium citrate. A tube containing EDTA was sampled first in order to determine full blood count and to exclude initial coagulation activation from venepuncture and venous stasis. The citrated WB was centrifuged at room temperature for 20 min at 1,500 g. The supernatant (4 mL of platelet poor plasma, PPP) was removed and depleted from albumin using HiTrap^®^ Blue HP columns for affinity chromatography. The resulting albumin-depleted plasma was stored at -70°C until use. In the second step, WB was collected in the same way as described above. After centrifugation, PPP was partly removed and replaced by the same volume of the autologous albumin-depleted plasma which was obtained in the first step. After gentle shaking ‘low albumin’ WB samples were obtained. WB samples containing physiological or ‘high’ levels of albumin were prepared by spiking ‘low albumin’ WB samples (300 μL) with increasing amounts (up to 30 μL) of HSA stock solution. Platelet function tests and thrombelastometry measurements were performed in the prepared WB samples. Subsequently, the remaining WB was centrifuged in order to prepare PPP samples. Thrombin generation, F1+2, and albumin levels were evaluated in PPP samples.

### Evaluation of primary haemostasis

Using the PFA 200, primary haemostasis is simulated with an in vitro quantitative measurement of platelet adhesion and aggregation in WB. The system uses citrated WB (800 μl) that is aspired under high shear stress rates through an aperture cut into a membrane coated with collagen (a subendothelial protein generally believed to be the initial matrix for platelet attachment) and either ADP or epinephrine. In response to the local shear stress and the agonists in the membrane, platelets are activated, adhere to collagen in the membrane surrounding the aperture, and aggregate until a stable platelet plug occludes the blood flow through the aperture. This time period recorded by the instrument is designated as the closure time (CT), representing a measure of platelet-dependent haemostasis, in particular platelet activation, adherence, and aggregability [18].

### Whole blood platelet aggregation assay

WB aggregation assessments were performed using a Chrono-Log Whole Blood Aggregometer Model 590 from Probe & Go (Endingen, Germany), which is based on the impedance method [19]. Impedance aggregometry results are expressed as amplitude (or maximum aggregation) in ohm at six minutes after reagent addition and as lag time (or aggregation time) in seconds, the time interval until the onset of platelet aggregation. The rate of platelet aggregation is expressed as slope in ohm/min. Collagen (2 ***μ***g mL^*-1*^, final concentration), purchased from Probe & Go, was used as platelet agonist, as previously described [20].

### Platelet aggregation associated ATP release

The aggregability of platelets can be assessed by quantitative determination of ATP exocytosis [21]. WB samples containing low, physiological or high levels of albumin were centrifuged at 150 g for 12 min in order to obtain platelet rich plasma (PRP) samples. Platelet aggregation was induced by addition of collagen (2 μg mL^-1^, final concentration) to 500 μL of the PRP samples. After two minutes, the PRP samples were centrifuged at 1,500 g for two minutes and proteins in the supernatant were precipitated with 0.4 M perchloric acid. After centrifugation at 12,000 g 100 μL of the supernatant were neutralized by addition of 10–12 μL of 2 M K_2_CO_3_ at 4°C. The supernatant obtained after centrifugation was used for HPLC analysis (injection volume: 40 μL). Separation of adenine nucleotides was performed on a Hypersil ODS column (5 μm, 250 × 4 mm I.D., equipped with a precolumn; Thermo Electron Corp. Runcorn, Cheshire, UK) using a L-2200 autosampler, two L-2130 HTA pumps, and a L-2450 diode array detector (all VWR International, West Chester, PA, USA) as previously described [22]. Detector signals (absorbance at 254 nm) were recorded and the program EZchrom Elite (VWR) was used for data acquisition and analysis.

### Whole blood platelet adhesion/aggregation assay

Platelet adhesion was assessed using a Cone and Platelet Analyzer (CPA) (DiaMed, Linz, Austria) as described previously [23]. Briefly, 130 μl of citrated WB was placed in polystyrene tubes and allowed to flow (1,300 s^-1^) for two minutes using a rotating Teflon cone. Subsequently, the wells were washed with PBS, stained with May-Grünwald solution and analyzed with an image analysis system. Surface coverage (SC) and average size (AS) were determined to elucidate platelet function. SC, representing platelet adhesion, is expressed as the percentage of total area covered by platelets. AS, representing platelet aggregation, is defined as the average size of the surface bound objects.

### Automated fluorogenic measurement of thrombin generation

Thrombin generation curves were monitored using calibrated automated thrombography (CAT) (Thrombinoscope BV, Maastricht, the Netherlands) [24]. The ability of a plasma sample to generate thrombin was assessed with respect to lag time preceding the thrombin burst (Lag Time), time to peak (ttPeak), peak height (Peak), maximum velocity of thrombin formation (VelIndex) and endogenous thrombin potential (ETP), and the time point of free thrombin disappearance (StartTail). Measurements were carried out in the presence of five pM of TF (final concentration). Measuring the formation of thrombin, the pivotal enzyme in haemostasis, has been shown to be an appropriate method to assess the coagulability of a given plasma sample [24, 25].

### Plasma levels of F1+2

Coagulation was triggered by addition of 40 μL of ‘trigger solution’ (0.35 pM TF, 3 mM CaCl_**2**_, 8 mM GPRP, final concentrations) to 300 μL of citrated PPP containing low, physiological, or high levels of albumin. After six minutes plasma levels of F1+2 were determined using ELISA kits from Behring Diagnostics GmbH (Marburg) as described in detail previously [26, 27].

### Whole blood tissue factor-triggered TEM assay

The clot formation process was monitored using the TEM coagulation analyser (ROTEM^®^05) from Matel Medizintechnik (Graz, Austria). The period of time from adding trigger to initial fibrin formation is designated as the ‘Coagulation time’ (CT); the time until the amplitude reaches 20 mm refers to the ‘Clot formation time’ (CFT). ‘Maximum clot firmness’ (MCF) reflects clot stability and the ‘alpha angle’ indicates the velocity of fibrin built-up and cross-linking. The final sample volume was 340 μL. Clot formation was initiated by addition of 40 μL of ‘trigger solution’ (without GPRP) to 300 μL of citrated WB. This method has been described in detail previously by Sorensen *et al*. [15].

### Standard laboratory tests

Plasma levels of FII, FVII, and FVIII were determined on a BM/Hitachi 917 from Roche (Vienna). Haematocrit and blood cell counts were determined on a Sysmex KX-21 N Automated Haematology Analyzer from Sysmex (Illinois, USA).

### Statistics

Differences in groups (low, physiological, and high albumin) were determined by means of one-way ANOVA for repeated measurements with Bonferroni’s multiple comparison test (using the GraphPad 5.0 Prism package). Paired t-test was used to evaluate platelet adhesion measurements on the CPA. All p-values of ≤ 0.05 were considered statistically significant. *… p ≤ 0.05, **… p ≤ 0.01, ***… p ≤ 0.001.

## Results

Coagulation factors, haematocrit, and blood cell counts of all subjects were in the physiological range of healthy adults [28]. The albumin concentration was 19.3 ± 7.7 g/L (‘low albumin’), 45.2 ± 7.8 g/L (‘physiological albumin’), and 67.5 ± 18.1 g/L (‘high albumin’), respectively. Plasma levels of coagulation factors in the prepared WB samples were approx. 80% of the respective physiological values due to losses during adsorption chromatography.

### Effects of different albumin concentrations on primary haemostasis

Primary haemostasis, assessed by means of the PFA 200 utilizing membranes coated with collagen/epinephrine, was significantly attenuated in the presence of increasing levels of albumin (**Table 1**): Closure times were significantly prolonged with increasing concentrations of albumin. However, this effect was not observed using membranes coated with collagen/ADP.

**Table 1:** Effects of low, physiologic, and high albumin concentrations on primary haemostasis.

	Low albumin	Phys. albumin	High albumin	Δ phys.→low	Δ phys.→high
Closure time (s)	167 ± 40	203 ± 44	248 ± 49	-35 ± 10, **	+45 ± 15, *
(coll./epinephrine)					
Closure time (s)	175 ± 64	192 ± 82	222 ± 87	-17 ± 10, n.s.	+30 ± 11, n.s.
(coll./ADP)					

Data were analysed by using one-way ANOVA for repeated measurements with Bonferroni’s multiple comparison test and are presented as mean ± SD, n = 10. Changes between baseline (physiologic albumin levels) and low or high albumin levels have been calculated for each individual. The respective mean changes with standard errors are presented.

*p < 0.05.

**p < 0.01.

### Effects of different albumin concentrations on platelet aggregation and ATP exocytosis

Platelet aggregation was assessed in ten individuals by impedance measurements and was significantly higher in the presence of low amounts of albumin compared with physiological amounts of albumin (**Table 2**). No significant differences were found between the physiological and the high albumin group. ATP measurements were performed in plasma from six individuals. The very conservative Bonferroni test indicated no significant difference between the separate groups, however, ANOVA shows an overall significant decrease of ATP release from low to high albumin concentrations.

**Table 2:** Effects of low, physiologic, and high albumin concentrations on platelet aggregation.

	Low albumin	Phys. albumin	High albumin	Δ phys.→low	Δ phys.→high
Amplitude (ohm)	8.7 ± 3.4	6.0 ± 3.1	4.9 ± 2.8	+2.5 ± 0.5, ***	-1.2 ± 0.3, n.s.
Slope (ohm/min)	4.9 ± 2.1	4.0 ± 1.9	3.5 ± 1.4	+0.9 ± 0.4, *	-0.5 ± 0.2, n.s.
Lag time (s)	100 ± 29	149 ± 63	178 ± 68	-49 ± 12, **	+29 ± 9, n.s.
ATP release	1.18 ± 0.14	1.02 ± 0.27	0.96 ± 0.13	+0.17 ± 0.04, n.s.	-0.06 ± 0.05, n.s.
(nmol/10^8^ cells)					

Data were analysed by using one-way ANOVA for repeated measurements with Bonferroni’s multiple comparison test and are presented as mean ± SD. Changes between baseline (physiologic albumin levels) and low or high albumin levels have been calculated for each individual. The respective mean changes with standard errors are presented.

*p < 0.05.

**p < 0.01.

***p < 0.001.

### Effects of different albumin concentrations on platelet adhesion/aggregation using CPA

Under the applied experimental conditions, albumin had no effect on platelet adhesion. The SC percentages were similar in all three groups (p = 0.4254, **Table 3**). Nonetheless, albumin affected platelet aggregation. The AS of WB samples containing low amounts of albumin was significantly higher than that of WB samples containing physiological levels of albumin (Table 3). However, no significant differences with respect to AS were found between the physiological and the high albumin group.

**Table 3:** Effects of low, physiologic, and high albumin concentrations on platelet adhesion/aggregation assessed by means of CPA.

	Low albumin	Phys. albumin		High albumin	Δ phys. → low	Δ phys. → high
SC (%)	11.7 ± 4.5		11.9 ± 3.1	10.5 ± 3.3	-0.18 ± 1.31, n.s.	-1.37 ± 1.16, n.s
AS (μm^2^)	52.9 ± 22.2		35.6 ± 9.1	35.8 ± 11.6	+17.3 ± 3.8, **	+0.2 ± 2.3, n.s.

Data were analysed by using one-way ANOVA for repeated measurements with Bonferroni’s multiple comparison test and are presented as mean ± SD. Changes between baseline (physiologic albumin levels) and low or high albumin levels have been calculated for each individual. The respective mean changes with standard errors are presented, n = 15.

**p < 0.01.

### Effects of different albumin concentrations on thrombin generation

CAT measurements predominantly indicated a tendency towards decreased thrombin formation comparing the physiological with the low albumin group (**Table 4**). Lag times were significantly prolonged and (thrombin) Peaks were significantly lower in the low albumin group. However, no significant differences were found between the physiological albumin group and the high albumin group. Moreover, ETP, the total amount of thrombin generated in the plasma sample, was not affected by albumin. Plasma levels of F1+2, another marker of thrombin generation, also indicated procoagulant action of albumin. Plasma levels of F1+2 were significantly higher in the physiological albumin group compared with the low albumin group (Table 4). However, no significant differences were found between the physiological and the high albumin group.

**Table 4:** Effects of low, physiologic, and high albumin concentrations on thrombin generation assessed by means of CAT and by F1+2 plasma levels.

	Low albumin	Phys. albumin	High albumin	Δ phys.→low	Δ phys.→high
Lag time (min)	2.9 ± 0.5	2.5 ± 0.3	2.4 ± 0.2	+0.4 ± 0.1, **	-0.2 ± 0.1, n.s.
Peak (nmol/L)	150 ± 33	185 ± 28	205 ± 32	-35 ± 6, ***	+20 ± 5, n.s.
ETP (nM.min)	1374 ± 128	1352 ± 261	1377 ± 247	+22 ± 51, n.s.	+25 ± 38, n.s.
F1+2 (pmol/L)	1095 ± 237	1254 ± 276	1325 ± 383	-159 ± 48, *	+71 ± 9, n.s.

Data were analysed by using one-way ANOVA for repeated measurements with Bonferroni’s multiple comparison test and are presented as mean ± SD, n = 10. Changes between baseline (physiologic albumin levels) and low or high albumin levels have been calculated for each individual. The respective mean changes with standard errors are presented.

*p < 0.05.

**p < 0.01

***p < 0.001.

### Effects of different albumin concentrations on thrombelastometry values

TEM values indicated anticoagulatory action of albumin (Table 5). MCFs and alpha angles significantly decreased in the presence of increasing albumin levels (low albumin group vs. physiological albumin group and physiological albumin group vs. high albumin group), indicating impaired clot formation. Moreover, CTs and CFTs were significantly prolonged in the high albumin group compared to the physiological albumin group, also indicating impaired clot formation. However, CTs and CFTs were not significantly different between the low albumin group and the physiological albumin group.

**Table 5:** Effects of low, physiologic, and high albumin concentrations on thrombelastometry values.

	Low albumin	Phys. albumin	High albumin	Δ phys.→low	Δ phys.→high
CT (s)	165 ± 41	172 ± 30	191 ± 45	-7 ± 6, n.s.	+19 ± 6, *
CFT (s)	169 ± 80	206 ± 83	268 ± 122	-37 ± 11, n.s	+61 ± 13, **
MCF mm)	51± 5	48 ± 6	46 ± 6	+3 ± 1, **	-2 ± 0.5, **
alpha (°)	62 ± 8	58 ± 8	51 ± 9	+4 ± 1, *	-7 ± 1, ***

Data were analysed by using one-way ANOVA for repeated measurements with Bonferroni’s multiple comparison test and are presented as mean ± SD, n = 15. Changes between baseline (physiologic albumin levels) and low or high albumin levels have been calculated for each individual. The respective mean changes with standard errors are presented.

*p < 0.05.

**p < 0.01.

***p < 0.001.

## Discussion

In the present study we show significant anticoagulant properties of albumin using several functional coagulation tests. Prolonged CTs, evaluated by means of the PFA 200 using membranes coated with collagen/epinephrine, indicate attenuated primary haemostasis with increasing albumin levels. Presumably, albumin, due to its ability to bind to epinephrine, coats the membran’s surface and thus inhibits the deposition of platelets [29]. Moreover, in WB samples with low concentrations of albumin impedance aggregometry as well as CPA measurements indicate enhanced platelet aggregation compared to WB containing physiological levels of albumin. TEM measurements indicate impaired clot formation with increasing albumin concentration. However, the absolute numbers of the changes of the coagulation parameters were, although significant, relatively small. Therefore, albumin has to be considered as mild but yet significant anticoagulant. However, thrombin peak formation was decreased in plasma samples containing low levels of albumin compared with the physiological albumin group. This is controversial to the results found in WB samples (PFA, platelet aggregation, CPA, and TEM).

The underlying mechanisms by which albumin attenuates platelet function in WB samples are not fully understood so far. However, various conclusions can be drawn from previous studies using washed platelets in artificial systems or from animal studies. In these studies the inhibitory effect of albumin on platelet aggregation could be partly attributed to its capability to bind arachidonic acid. As a consequence, platelet-derived cyclooxygenases are deprived of their substrate and can’t form the platelet agonist thromboxane A2. Additionally, albumin has been shown to inactivate thromboxane A2 directly through binding [30]. Moreover, it has also been reported that albumin can bind platelet-activating factor (PAF) with high affinity [31]. As a result, PAF-induced platelet aggregation is suppressed in the presence of increasing levels of albumin in a concentration-dependent manner [32].

Another mechanism through which albumin can exert its anti-aggregatory action in WB samples might be its capability to concentration-dependently induce inducible nitric oxide synthase in macrophages [33, 34]. This induction would lead to enhanced formation of the potent platelet inhibitor nitric oxide (NO).

Binding of the platelet aggregation inhibitor prostacyclin (PGI_2_) to albumin prevents its degradation [35]. Increased binding of PGI_2_ at higher albumin concentrations could further trigger the anti-aggregatory effects. Furthermore, the availability of PGI_2_ for its receptor on platelets has been shown to increase with augmenting albumin levels [36].

Regarding the effects of albumin on secondary haemostasis, evaluated in terms of thrombin generation, the results presented herein are contradictory to previous studies. A heparin-like (and thus anticoagulant) activity of albumin has been suggested [9]. We, however, found accelerated formation of thrombin associated with high thrombin peaks and markedly elevated levels of F1+2 in the presence of increasing amounts of albumin in TF-activated PPP samples. This apparent procoagulant action of albumin, however, cannot be attributed to the presence of the previously described “procoagulant albumin”. Procoagulant albumin has been shown to support coagulation by inducing TF expression in endothelial cells and monocytes but we measured thrombin generation in PPP samples lacking these cells [37]. To our knowledge, the underlying mechanisms of the procoagulant properties of albumin in PPP samples are missing so far. Whether albumin is capable of binding to coagulation inhibitors/activators and, in consequence, alter their functionality is conceivable but still lacks experimental data. On the other hand, the procoagulant action of albumin presented herein might be of minor physiological or clinical significance. We found that albumin has no influence on ETP, a parameter frequently used to estimate the coagulability of plasma [24, 25].

It has been shown that TEM measurements allow a complete evaluation of the process of clot initiation, formation, and stability [15]. Using this method, we found that albumin clearly exerts anticoagulant properties. Prolonged clotting times (CTs and CFTs) in the high vs. the physiological albumin group and decreased MCFs and alpha angles in WB samples containing higher levels of albumin (low albumin group vs. physiological albumin group and physiological albumin group vs. high albumin group) indicated impaired clot formation capacity of WB samples. In particular, the observed changes of CFT and alpha angle values indicate a significant deceleration of fibrin built-up and decreasing MCFs indicate both, impaired fibrin built-up and impaired platelet function [38]. These findings are in good agreement with results from two recent studies. Pathirana *et al*. have shown that a 40% haemodilution with a 4% albumin solution impaired coagulation to a greater extent than an equivalent haemodilution with saline [39]. The present study shows albumin-induced impairment of coagulation even at markedly lower degrees of haemodilution (~10%). Presumably, the high sensitivity of our measurements is attributable to the low amounts of trigger (TF is in the picomolar range) applied to initiate coagulation [15]. Thölking *et al*. have also shown impaired coagulation after the use of 5% HSA solution in therapeutic plasma exchange [40]. However, their findings cannot directly be compared with the present study. The coagulopathy found in their study is mainly attributable to depletion of procoagulatory factors, e.g. approx. 50% reduced fibrinogen levels due to infusion of the HSA solution.

The exact mechanisms by which albumin impairs coagulation in its entirety have yet to be elucidated. However, results from previous studies as well as experimental data obtained in this study reveal that albumin can interact with fibrinogen resulting in impaired fibrinogen activity [41]. Consistently, it has been shown that albumin-induced coagulopathy can effectively be reversed by addition of fibrinogen concentrate [42].

The findings of the present study might help to partially explain thrombotic events in several clinical situations associated with low serum albumin levels [16, 17]. The authors acknowledge that it is currently not clear whether serum albumin is mechanistically involved or whether this correlation is a manifestation of underlying inflammatory processes. The results from our study are derived from healthy persons, and, thus, cannot directly be transferred to the clinical situation. However, our results would suggest that low serum albumin levels contribute to VTE. WB samples containing low amounts of albumin were in a hypercoagulable state compared to WB samples containing physiological levels of albumin. Most likely, low levels of serum albumin, as observed in cancer patients, slightly shift the haemostatic system towards hypercoagulability, a prerequisite for the development of VTE.

It has been shown that cirrhotic patients with ascites benefit from albumin therapy by preventing postparacentesis circulatory dysfunction [7]. In contrast, liver disease depending on coexisting circumstantial risk factors has been shown to be associated with a derangement of haemostasis easily leading to bleeding or thrombosis [43]. Albumin, by virtue of its anticoagulatory properties shown herein, might be such a risk factor. Albumin therapy in liver disease patients might therefore be associated with risk of hemorrhage.

Finally, the results presented herein help to explain the well-functioning haemostasis in neonates despite their low levels of coagulation factors [26, 44]. In the last years several features have already been shown contributing to the procoagulant readiness of neonatal blood, e.g. unusually large von Willebrand factor multimers [45] and physiological low levels of natural anticoagulants [26]. We propose that the low physiological levels of albumin (approx. 10 g/L during the first 12 weeks) [46] are an additional issue. According to our present findings, these low albumin levels might contribute to efficient platelet aggregation and clot formation in neonates.

It has to be stated that albumin might have a limited effect on the coagulation system in the clinical setting. Rasmussen et al. have shown that albumin reduced the coagulation competence and increased hemorrhage during major non cardiac surgery but did not reveal significant clinical impact [11].

## Supporting information

S1 File Quantitative summary of the research data presented in Tables 1–5. (PDF)

## Abbreviations

CAT: calibrated automated thrombography
CFT: clot formation time
CPA: cone and platelet analyser
CT: coagulation time
eNOS: endothelial nitric oxide synthase
ETP: endogenous thrombin potential
F 1+2: prothrombin fragment 1+2
Hct: haematocrit
HSA: human serum albumin
MCF: maximum clot firmness
NO: nitric oxide
PAF: platelet-activating factor
PFA: platelet function analyser
PGI_**2**_: prostacyclin
PPP: platelet poor plasma
PRP: platelet rich plasma
SD: standard deviation
TEM: thrombelastometry
TF: tissue factor
VELINDEX: peak rate of thrombin generation
VTE: venous thromboembolism
WB: whole blood

## References

[1] Nicholson JP, Wolmarans MR, Park GR. The role of albumin in critical illness. Br J Anaesth. 2000;85(4):599–610. . 11064620 10.1093/bja/85.4.599

[2] Oettl K, Stauber RE. Physiological and pathological changes in the redox state of human serum albumin critically influence its binding properties. Br J Pharmacol. 2007;151(5):580–90. doi: 10.1038/sj.bjp.0707251 ; PubMed Central PMCID: PMCPMC2013999. 17471184 10.1038/sj.bjp.0707251 PMC2013999

[3] Becker BF, Jacob M, Leipert S, Salmon AH, Chappell D. Degradation of the endothelial glycocalyx in clinical settings: searching for the sheddases. Br J Clin Pharmacol. 2015;80(3):389–402. doi: 10.1111/bcp.12629 ; PubMed Central PMCID: PMCPMC4574825. 25778676 10.1111/bcp.12629 PMC4574825

[4] Jellinge ME, Henriksen DP, Hallas P, Brabrand M. Hypoalbuminemia is a strong predictor of 30-day all-cause mortality in acutely admitted medical patients: a prospective, observational, cohort study. PLoS One. 2014;9(8):e105983 doi: 10.1371/journal.pone.0105983 ; PubMed Central PMCID: PMCPMC4141840. 25148079 10.1371/journal.pone.0105983 PMC4141840

[5] Finestone HM, Greene-Finestone LS, Wilson ES, Teasell RW. Prolonged length of stay and reduced functional improvement rate in malnourished stroke rehabilitation patients. Arch Phys Med Rehabil. 1996;77(4):340–5. . 8607756 10.1016/s0003-9993(96)90081-7

[6] Golub R, Sorrento JJ Jr., Cantu R Jr., Nierman DM, Moideen A, Stein HD. Efficacy of albumin supplementation in the surgical intensive care unit: a prospective, randomized study. Crit Care Med. 1994;22(4):613–9. . 8143470 10.1097/00003246-199404000-00017

[7] Gines A, Fernandez-Esparrach G, Monescillo A, Vila C, Domenech E, Abecasis R, et al Randomized trial comparing albumin, dextran 70, and polygeline in cirrhotic patients with ascites treated by paracentesis. Gastroenterology. 1996;111(4):1002–10. . 8831595 10.1016/s0016-5085(96)70068-9

[8] Suarez JI, Martin RH, Hohmann SF, Calvillo E, Bershad EM, Venkatasubba Rao CP, et al Human Albumin Use in Adults in U.S. Academic Medical Centers. Crit Care Med. 2016 doi: 10.1097/CCM.0000000000002010 . 27632679 10.1097/CCM.0000000000002010

[9] Joorgensen KA, Stoffersen E. Heparin like activity of albumin. Thromb Res. 1979;16(3–4):569–74. . 516011 10.1016/0049-3848(79)90105-1

[10] Jorgensen KA, Stoffersen E. On the inhibitory effect of albumin on platelet aggregation. Thromb Res. 1980;17(1–2):13–8. . 6990546 10.1016/0049-3848(80)90289-3

[11] Rasmussen KC, Hojskov M, Johansson PI, Kridina I, Kistorp T, Salling L, et al Impact of Albumin on Coagulation Competence and Hemorrhage During Major Surgery: A Randomized Controlled Trial. Medicine (Baltimore). 2016;95(9):e2720 doi: 10.1097/MD.0000000000002720 ; PubMed Central PMCID: PMCPMC4782842. 26945358 10.1097/MD.0000000000002720 PMC4782842

[12] Skhirtladze K, Base EM, Lassnigg A, Kaider A, Linke S, Dworschak M, et al Comparison of the effects of albumin 5%, hydroxyethyl starch 130/0.4 6%, and Ringer's lactate on blood loss and coagulation after cardiac surgery. Br J Anaesth. 2014;112(2):255–64. doi: 10.1093/bja/aet348 . 24169821 10.1093/bja/aet348

[13] Liu L, Murray DK, Dameron CT, Parker CJ, Rodgers GM. Biochemical characterization of procoagulant albumin. Thromb Res. 1997;85(5):399–411. . 9076897 10.1016/s0049-3848(97)00028-5

[14] McCammon AT, Wright JP, Figueroa M, Nielsen VG. Hemodilution with albumin, but not Hextend, results in hypercoagulability as assessed by Thrombelastography in rabbits: role of heparin-dependent serpins and factor VIII complex. Anesth Analg. 2002;95(4):844–50, table of contents. . 12351255 10.1097/00000539-200210000-00010

[15] Sorensen B, Johansen P, Christiansen K, Woelke M, Ingerslev J. Whole blood coagulation thrombelastographic profiles employing minimal tissue factor activation. J Thromb Haemost. 2003;1(3):551–8. . 12871465 10.1046/j.1538-7836.2003.00075.x

[16] Konigsbrugge O, Posch F, Riedl J, Reitter EM, Zielinski C, Pabinger I, et al Association Between Decreased Serum Albumin With Risk of Venous Thromboembolism and Mortality in Cancer Patients. Oncologist. 2016;21(2):252–7. doi: 10.1634/theoncologist.2015-0284 ; PubMed Central PMCID: PMCPMC4746083. 26764252 10.1634/theoncologist.2015-0284 PMC4746083

[17] Folsom AR, Lutsey PL, Heckbert SR, Cushman M. Serum albumin and risk of venous thromboembolism. Thromb Haemost. 2010;104(1):100–4. doi: 10.1160/TH09-12-0856 ; PubMed Central PMCID: PMCPMC2902783. 20390234 10.1160/TH09-12-0856 PMC2902783

[18] Chang YW, Liao CH, Day YJ. Platelet function analyzer (PFA-100) offers higher sensitivity and specificity than thromboelastography (TEG) in detection of platelet dysfunction. Acta Anaesthesiol Taiwan. 2009;47(3):110–7. . 19762300 10.1016/s1875-4597(09)60036-9

[19] Morel-Kopp MC, Tan CW, Brighton TA, McRae S, Baker R, Tran H, et al Validation of whole blood impedance aggregometry as a new diagnostic tool for HIT: results of a large Australian study. Thromb Haemost. 2012;107(3):575–83. doi: 10.1160/TH11-09-0631 . 22234599 10.1160/TH11-09-0631

[20] Cvirn G, Gallistl S, Rehak T, Jurgens G, Muntean W. Elevated thrombin-forming capacity of tissue factor-activated cord compared with adult plasma. J Thromb Haemost. 2003;1(8):1785–90. . 12911594 10.1046/j.1538-7836.2003.00320.x

[21] von Papen M, Gambaryan S, Schutz C, Geiger J. Determination of ATP and ADP Secretion from Human and Mouse Platelets by an HPLC Assay. Transfus Med Hemother. 2013;40(2):109–16. doi: 10.1159/000350294 ; PubMed Central PMCID: PMCPMC3638993. 23652982 10.1159/000350294 PMC3638993

[22] Deak AT, Blass S, Khan MJ, Groschner LN, Waldeck-Weiermair M, Hallstrom S, et al IP3-mediated STIM1 oligomerization requires intact mitochondrial Ca2+ uptake. J Cell Sci. 2014;127(Pt 13):2944–55. doi: 10.1242/jcs.149807 PubMed Central PMCID: PMCPMC4077590. 24806964 10.1242/jcs.149807 PMC4077590

[23] Varon D, Dardik R, Shenkman B, Kotev-Emeth S, Farzame N, Tamarin I, et al A new method for quantitative analysis of whole blood platelet interaction with extracellular matrix under flow conditions. Thromb Res. 1997;85(4):283–94. . 9062952 10.1016/s0049-3848(97)00014-5

[24] Al Dieri R, de Laat B, Hemker HC. Thrombin generation: what have we learned? Blood Rev. 2012;26(5):197–203. doi: 10.1016/j.blre.2012.06.001 . 22762893 10.1016/j.blre.2012.06.001

[25] Mann KG, Orfeo T, Butenas S, Undas A, Brummel-Ziedins K. Blood coagulation dynamics in haemostasis. Hamostaseologie. 2009;29(1):7–16. ; PubMed Central PMCID: PMCPMC3152749. 19151839 PMC3152749

[26] Cvirn G, Gallistl S, Leschnik B, Muntean W. Low tissue factor pathway inhibitor (TFPI) together with low antithrombin allows sufficient thrombin generation in neonates. J Thromb Haemost. 2003;1(2):263–8. . 12871499 10.1046/j.1538-7836.2003.00081.x

[27] Cvirn G, Schlagenhauf A, Leschnik B, Koestenberger M, Roessler A, Jantscher A, et al Coagulation changes during presyncope and recovery. PLoS One. 2012;7(8):e42221 doi: 10.1371/journal.pone.0042221 ; PubMed Central PMCID: PMCPMC3410921. 22876309 10.1371/journal.pone.0042221 PMC3410921

[28] Andrew M, Vegh P, Johnston M, Bowker J, Ofosu F, Mitchell L. Maturation of the hemostatic system during childhood. Blood. 1992;80(8):1998–2005. . 1391957

[29] de Vera N, Cristofol RM, Rodriguez Farre E. Protein binding and stability of norepinephrine in human blood plasma. Involvement of prealbumin, alpha 1-acid glycoprotein and albumin. Life Sci. 1988;43(16):1277–86. . 3172980 10.1016/0024-3205(88)90582-6

[30] Maclouf J, Kindahl H, Granstrom E, Samuelsson B. Interactions of prostaglandin H2 and thromboxane A2 with human serum albumin. Eur J Biochem. 1980;109(2):561–6. . 7408901 10.1111/j.1432-1033.1980.tb04828.x

[31] Clay KL, Johnson C, Henson P. Binding of platelet activating factor to albumin. Biochim Biophys Acta. 1990;1046(3):309–14. . 2171672 10.1016/0005-2760(90)90246-t

[32] Grigoriadis G, Stewart AG. Albumin inhibits platelet-activating factor (PAF)-induced responses in platelets and macrophages: implications for the biologically active form of PAF. Br J Pharmacol. 1992;107(1):73–7. ; PubMed Central PMCID: PMCPMC1907628. 1330167 10.1111/j.1476-5381.1992.tb14465.x PMC1907628

[33] Poteser M, Wakabayashi I. Serum albumin induces iNOS expression and NO production in RAW 267.4 macrophages. Br J Pharmacol. 2004;143(1):143–51. doi: 10.1038/sj.bjp.0705897 ; PubMed Central PMCID: PMCPMC1575263. 15289288 10.1038/sj.bjp.0705897 PMC1575263

[34] Podesser BK, Hallstrom S. Nitric oxide homeostasis as a target for drug additives to cardioplegia. Br J Pharmacol. 2007;151(7):930–40. doi: 10.1038/sj.bjp.0707272 ; PubMed Central PMCID: PMCPMC2042932. 17486142 10.1038/sj.bjp.0707272 PMC2042932

[35] Weiss HJ, Turitto VT. Prostacyclin (prostaglandin I2, PGI2) inhibits platelet adhesion and thrombus formation on subendothelium. Blood. 1979;53(2):244–50. . 367465

[36] Tsai AL, Hsu MJ, Patsch W, Wu KK. Regulation of PGI2 activity by serum proteins: serum albumin but not high density lipoprotein is the PGI2 binding and stabilizing protein in human blood. Biochim Biophys Acta. 1991;1115(2):131–40. . 1764464 10.1016/0304-4165(91)90021-8

[37] Faucette KJ, Parker CJ, McCluskey T, Bernshaw NJ, Rodgers GM. Induction of tissue factor activity in endothelial cells and monocytes by a modified form of albumin present in normal human plasma. Blood. 1992;79(11):2888–95. . 1586736

[38] Lang T, Toller W, Gutl M, Mahla E, Metzler H, Rehak P, et al Different effects of abciximab and cytochalasin D on clot strength in thrombelastography. J Thromb Haemost. 2004;2(1):147–53. . 14717978 10.1111/j.1538-7836.2004.00555.x

[39] Pathirana S, Wong G, Williams P, Yang K, Kershaw G, Dunkley S, et al The effects of haemodilution with albumin on coagulation in vitro as assessed by rotational thromboelastometry. Anaesth Intensive Care. 2015;43(2):187–92. . 25735683 10.1177/0310057X1504300207

[40] Tholking G, Mesters R, Dittrich R, Pavenstadt H, Kumpers P, Reuter S. Assessment of Hemostasis after Plasma Exchange Using Rotational Thrombelastometry (ROTEM). PLoS One. 2015;10(6):e0130402 doi: 10.1371/journal.pone.0130402 ; PubMed Central PMCID: PMCPMC4488284. 26121484 10.1371/journal.pone.0130402 PMC4488284

[41] Galanakis DK. Anticoagulant albumin fragments that bind to fibrinogen/fibrin: possible implications. Semin Thromb Hemost. 1992;18(1):44–52. doi: 10.1055/s-2007-1002409 . 1574716 10.1055/s-2007-1002409

[42] Winstedt D, Hanna J, Schott U. Albumin-induced coagulopathy is less severe and more effectively reversed with fibrinogen concentrate than is synthetic colloid-induced coagulopathy. Scand J Clin Lab Invest. 2013;73(2):161–9. doi: 10.3109/00365513.2012.762114 . 23368782 10.3109/00365513.2012.762114

[43] Kujovich JL. Coagulopathy in liver disease: a balancing act. Hematology Am Soc Hematol Educ Program. 2015;2015:243–9. doi: 10.1182/asheducation-2015.1.243 . 26637729 10.1182/asheducation-2015.1.243

[44] Cvirn G, Koestenberger M, Leschnik B, Male C, Kutschera J, Ferstl U, et al Protein S modulates the anticoagulant action of recombinant human activated protein C: a comparison between neonates and adults. Br J Pharmacol. 2005;146(8):1082–6. doi: 10.1038/sj.bjp.0706436 ; PubMed Central PMCID: PMCPMC1751238. 16273121 10.1038/sj.bjp.0706436 PMC1751238

[45] Rehak T, Cvirn G, Gallistl S, Leschnik B, Kostenberger M, Katzer H, et al Increased shear stress- and ristocetin-induced binding of von Willebrand factor to platelets in cord compared with adult plasma. Thromb Haemost. 2004;92(4):682–7. doi: 10.1160/TH04-05-0270 . 15467896 10.1160/TH04-05-0270

[46] Krauer B, Dayer P, Anner R. Changes in serum albumin and alpha 1-acid glycoprotein concentrations during pregnancy: an analysis of fetal-maternal pairs. Br J Obstet Gynaecol. 1984;91(9):875–81. . 6477846 10.1111/j.1471-0528.1984.tb03700.x

