# Supplementary material for: Anticoagulant action of low, physiologic, and high albumin levels in whole blood
Source: PLoS One. 2017 Aug 11;12(8):e0182997. doi: 10.1371/journal.pone.0182997 (PMC5553770; doi:10.1371/journal.pone.0182997)
Supplement: S1 File — (PDF) [file pone.0182997.s001.pdf]

S1 File : Data availability

PFA 200, Closure times; Collagen/Epinephrine:

Low albumin	Phys. Albumin	High albumin
151	163	187
133	186	173
174	189	290
133	190	245
183	285	265
200	228	273
218	253	300
227	219	297
113	136	268
141	176	182

PFA 200, Closure times (s); Collagen/ADP:

Low albumin	Phys. Albumin	High albumin
165	190	185
122	108	139
173	188	300
199	299	299
302	300	300
170	197	227
214	235	300
94	93	101
141	123	148
166	187	217

Impedance aggregometry; Amplitude (ohm)

Low albumin	Phys. Albumin	High albumin
10	8	7
5	4	3
7	6	5
4	2	1
11	7	7
8	7	7
4	1	0
13	7	4
13	10	8
11	9	7

Impedance aggregometry; Slope (ohm/min)

Low albumin	Phys. Albumin	High albumin
6	6	5
3	3	2
3	3	3
2	1	1
5	4	4
5	4	4
3	2	2
8	4	4
8	7	5
6	6	5

Impedance aggregometry; Lag Time (s)

Low albumin	Phys. Albumin	High albumin
82	96	121
100	124	165
68	120	114
125	197	260
76	122	132
85	100	103
158	300	300
133	187	235
88	124	150
86	124	202

ATP release (nmol/10^8 cells)

Low albumin	Phys. Albumin	High albumin
1,17	1,1	1,03
1,23	1,17	0,94
1,22	0,98	0,91
0,99	0,7	0,88
1,3	1,18	1,08
1,19	0,97	0,89

CPA; Surface coverage (%)

Low albumin	Physiologic alb	High albumin
8	17	7,4
7,1	9	5,5
12	13	12
13	19	11
16	15	11,1
8,6	12	12
10	12	14
10	11	8
9	12	10
8,5	10	6
8,1	10	6,5
12	9	10
14	8	12
20	11	16
19	10	16

CPA; Average size (µm2)

Low albumin	Physiologic alb	High albumin
66	52	38
45	43	39
79	49	32
75	48	58
105	45	63
49	32	31
42	35	32
33	27	24
32	31	29
39	30	26
43	31	32
45	28	28
40	28	35
51	27	36
49	28	34

CAT; Lag Time (min)

Low albumin	Phys. Albumin	High albumin
3,44	2,89	2,67
3,44	2,33	2,33
3,89	2,89	2,67
2,33	2,22	2,33
2,67	2,33	2,33
2,78	2,67	2,33
2,67	2,67	2,33
2,33	2,00	2,00
2,89	2,67	2,33
2,95	2,53	2,38

CAT; Peak (nmol/L)

Low albumin	Phys. Albumin	High albumin
126	161	174
145	189	190
98	172	207
200	237	239
165	161	179
114	150	181
187	210	267
155	180	185
165	209	222
148	183	206

CAT; ETP (nM.min)

Low albumin	Phys. Albumin	High albumin
1386	1418	1340
1316	1352	1238
1464	1471	1668
1595	1939	1903
1397	1153	1189
1131	1064	1204
1357	1130	1348
1290	1257	1188
1432	1388	1315
1373	1351	1379

F1+2 (pmol/L)

Low albumin	Phys. Albumin	High albumin
1090	1238	1298
1340	1704	1754
909	985	1087
1218	1246	1301
1020	1073	1120
1330	1458	1512
1202	1525	1588
832	921	1018
1103	1513	1566
908	880	1004

TEM; Coagulation time (s)

Low albumin	Phys. Albumin	High albumin
169	188	188
143	158	166
148	159	169
183	185	186
219	191	193
174	163	175
193	181	192
244	213	243
124	125	132
164	182	214
124	136	149
139	188	212
160	205	301
162	169	198
136	142	154

TEM; Clot formation time (s)

Low albumin	Phys. Albumin	High albumin
119	155	201
87	123	138
232	241	289
165	206	205
288	228	263
185	246	261
153	207	255
302	357	510
141	148	174
115	136	205
152	170	215
158	274	440
213	319	423
109	146	241
115	139	197

TEM; Maximum Clot Firmness (mm)

Low albumin	Phys. Albumin	High albumin
47	42	41
48	46	46
46	50	47
47	45	46
53	47	47
46	40	39
54	53	53
52	50	47
49	48	46
47	42	39
48	37	35
58	56	50
53	50	48
54	54	50
56	53	49

TEM; alpha (°)

Low albumin	Phys. Albumin	High albumin
68	62	56
72	68	64
54	50	47
60	55	55
45	54	50
60	52	50
63	58	51
46	42	34
65	64	60
69	67	57
63	61	56
66	53	39
58	47	38
68	66	50
68	65	56
